# Anti-malarial prescription practices among children admitted to six public hospitals in Uganda from 2011 to 2013

**DOI:** 10.1186/s12936-015-0851-8

**Published:** 2015-08-27

**Authors:** Asadu Sserwanga, David Sears, Bryan K. Kapella, Ruth Kigozi, Denis Rubahika, Sarah G. Staedke, Moses Kamya, Steven S. Yoon, Michelle A. Chang, Grant Dorsey, Arthur Mpimbaza

**Affiliations:** Infectious Diseases Research Collaboration, Kampala, Uganda; Department of Medicine, San Francisco General Hospital, University of California, San Francisco, USA; Malaria Branch, Division of Parasitic Diseases and Malaria, US Centers for Disease Control and Prevention, Atlanta, USA; National Malaria Control Programme, Ministry of Health Uganda, Kampala, Uganda; London School of Hygiene and Tropical Medicine, London, UK; Child Health and Development Centre, Makerere University College of Health Sciences, Kampala, Uganda

**Keywords:** Anti-malarial, Treatment, Children, Hospitals, Uganda

## Abstract

**Background:**

In 2011, Uganda’s Ministry of Health switched policy from presumptive treatment of malaria to recommending parasitological diagnosis prior to treatment, resulting in an expansion of diagnostic services at all levels of public health facilities including hospitals. Despite this change, anti-malarial drugs are often prescribed even when test results are negative. Presented is data on anti-malarial prescription practices among hospitalized children who underwent diagnostic testing after adoption of new treatment guidelines.

**Methods:**

Anti-malarial prescription practices were collected as part of an inpatient malaria surveillance program generating high quality data among children admitted for any reason at government hospitals in six districts. A standardized medical record form was used to collect detailed patient information including presenting symptoms and signs, laboratory test results, admission and final diagnoses, treatments administered, and final outcome upon discharge.

**Results:**

Between July 2011 and December 2013, 58,095 children were admitted to the six hospitals (hospital range 3294–20,426).A total of 56,282 (96.9 %) patients were tested for malaria, of which 26,072 (46.3 %) tested positive (hospital range 5.9–57.3 %). Among those testing positive, only 84 (0.3 %) were first tested after admission and 295 of 30,389 (1.0 %) patients who tested negative at admission later tested positive. Of 30,210 children with only negative test results, 11,977 (39.6 %) were prescribed an anti-malarial (hospital range 14.5–53.6 %). The proportion of children with a negative test result who were prescribed an anti-malarial fluctuated over time and did not show a significant trend at any site with the exception of one hospital where a steady decline was observed. Among those with only negative test results, children 6–12 months of age (aOR 3.78; p < 0.001) and those greater than 12 months of age (aOR 4.89; p < 0.001) were more likely to be prescribed an anti-malarial compared to children less than 6 months of age. Children with findings suggestive of severe malaria were also more likely to be prescribed an anti-malarial after a negative test result (aOR 1.98; p < 0.001).

**Conclusions:**

Despite high testing rates for malaria at all sites, prescription of anti-malarials to patients with negative test results remained high, with the exception of one site where a steady decline occurred.

## Background

Malaria is reported by the Ministry of Health’s Health Management Information System to be the leading cause of morbidity and mortality in Uganda, accounting for 30–50 % of outpatient visits, 35 % of hospital admissions, and 9–14 % of hospital deaths [1]. More recent estimates from the World Health Organization (WHO) listed Uganda as having one of the highest malaria burdens in the world with an estimated 10,338,093 probable and confirmed cases, 592,264 inpatient malaria cases, and 6585 inpatient deaths in 2012 [2]. For children admitted to the hospital with malaria, case management including early diagnosis and prompt, effective treatment is a key strategy for minimizing deaths [3].

In 2010, WHO introduced two major changes to treatment guidelines for malaria case management. First was the replacement of presumptive treatment with parasitological diagnosis whenever possible, and second was the replacement of intravenous quinine with intravenous artesunate as first-line treatment for severe malaria [4]. The Uganda Ministry of Health adopted both changes in 2011 [5]. The new emphasis on parasitological diagnosis does not distinguish between patients with uncomplicated and severe malaria. However, due to the life-threatening nature of severe malaria, the WHO emphasizes treatment should not be delayed in the interest of obtaining a test result [6]. Parasitological confirmation of malaria is essential for targeting anti-malarial treatment to appropriate patients [7, 8]. Advantages of targeted treatment include minimizing unnecessary use of drugs, avoiding selection of drug resistance, and reducing exposure of patients to adverse events associated with unnecessary anti-malarial drug treatments [9–11]. In the case of severe malaria, parasitological confirmation of disease minimizes misclassification of other severe febrile illnesses as malaria [6].

Despite changes in policy recommendations and reported benefits, reports indicate that anti-malarial therapy continues to be prescribed to children even following a negative malaria test result [12]. Utilizing data collected from an enhanced inpatient malaria surveillance system from July 2011 through December 2013, presented are results on anti-malarial prescription practices among children with test results admitted to six government hospitals in Uganda from areas of varying malaria endemicity. To improve the understanding of prescription practices, presented to are factors associated with the prescription of anti-malarials to children with negative malaria test results.

## Methods

### Uganda malaria surveillance project (UMSP)

**Fig. 1:**
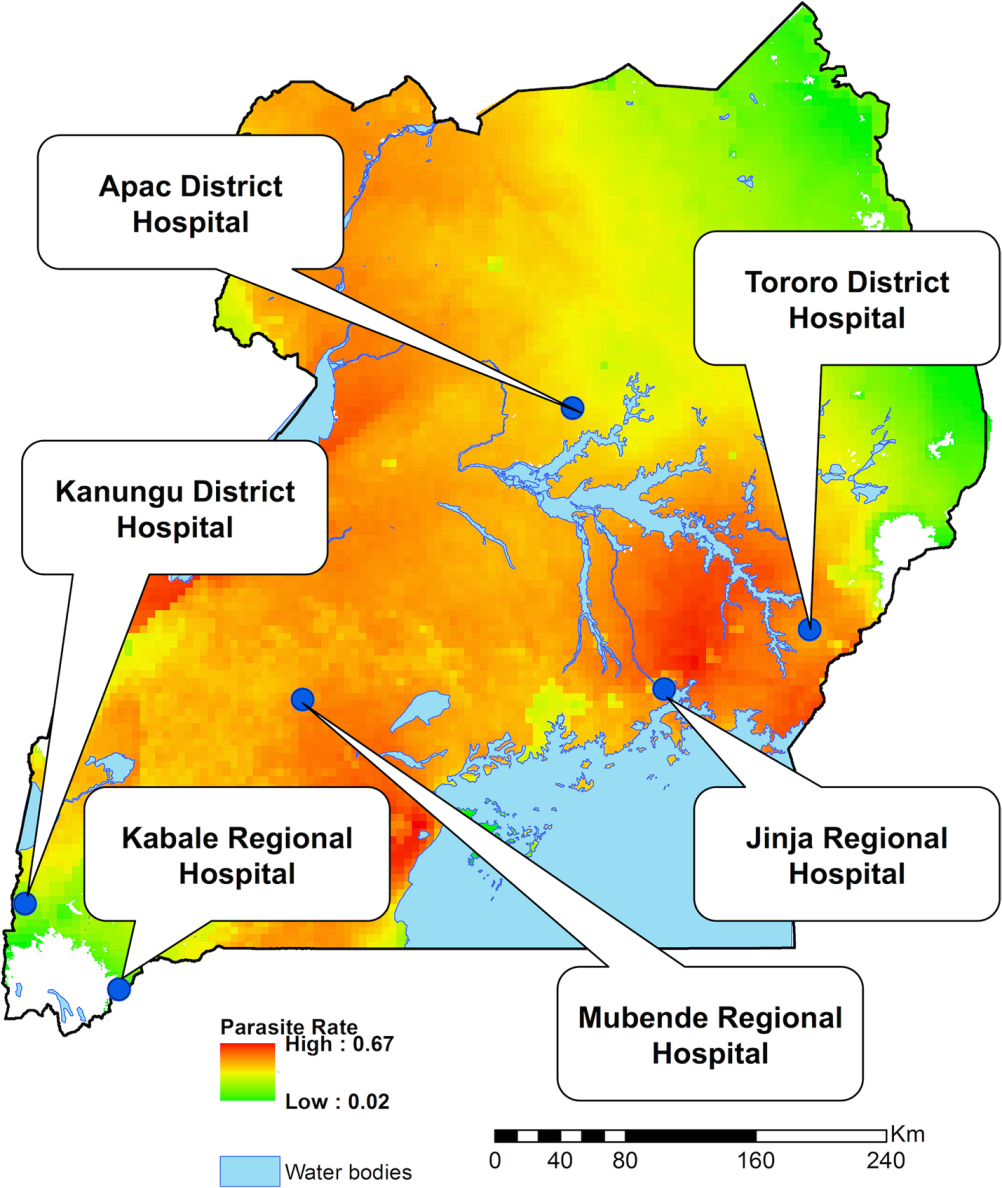
Hospital locations overlying map of Uganda with modelled parasite rates. The *colours* are PfPR in the over 2 and up to 10 year age group from the MAP 2010 data set [38]

Between April 2010 and June 2011, the Uganda Malaria Surveillance Project (UMSP) in collaboration with the National Malaria Control Programme (NMCP) established an inpatient malaria surveillance system at public hospitals in six districts—Tororo, Jinja, Kanungu, Mubende, Apac and Kabale—representing distinct geography and malaria transmission intensity (Fig. 1). UMSP has also been supporting outpatient malaria sentinel surveillance programme in these six districts since 2006 [13]. Tororo, Kambuga (Kanungu District), and Apac are general hospitals providing basic inpatient health services whereas Jinja and Kabale are regional referral hospitals offering specialist medical and surgical services. Mubende was elevated to a regional referral hospital in 2012.

A standardized individual medical record form (MRF) was developed by UMSP in consultation with the NMCP. The MRF collects detailed patient information including presenting symptoms and signs, laboratory test results, admission and final diagnoses, treatments administered, and final outcome upon discharge. The MRF uses check boxes to minimize transcription errors, and incorporates Integrated Management of Childhood Illnesses (IMCI) terminology for signs and symptoms, a standard framework familiar to most clinicians in Uganda. UMSP was responsible for ensuring that MRFs were available at all study sites.

### Training

Upon initiation of the inpatient malaria surveillance system, training was provided to the clinical staff on the use of the new MRF, the importance of good medical record keeping, the use of data to improve the quality of services, and proper case management of uncomplicated and severe malaria. Case management training was conducted with all clinicians during a 1-day session that was repeated on a second day to maximize the proportion of staff who received training. Through lectures and the distribution of job aides, case management training focused on the importance of malaria diagnosis based on parasitological confirmation, the use of quinine for severe malaria (prior to the policy change recommending intravenous artesunate), and strategies aimed at streamlining patient flow within the hospital with the goal of reducing wait time. Hospital staff were also trained to test all paediatric admissions for malaria as a component of the programme’s goal of measuring the test positivity rate of all inpatients. This training included political and administrative district leadership, hospital administration, and hospital staff.

Prior to start of the surveillance programme a laboratory staff at respective hospitals underwent a 3-day refresher course in malaria microscopy. The curriculum and training materials were developed by a team of laboratory experts from UMSP, Ministry of Health, and Central Public Health Laboratories as well as district laboratory focal persons, the details of which have been previously published [14]. Training at all sites was followed by a pilot period to identify and address additional barriers to collecting high quality data. Sites were allowed to proceed only when the proportion of paediatric admissions tested for malaria was greater than 90 %. For most sites, this occurred approximately 2 months after the start of the pilot.

### Patient care

Patient care in all hospitals except Jinja was provided by clinical officers (diploma level training in medicine) and nurses. A clinical officer made the decision for admission after the child was initially seen in the outpatient department. All admitted children had an MRF completed by the clinical officer, documenting the child’s presenting symptoms and signs, initial diagnoses, and treatments prescribed. For most hospitalized children, nurses provided routine care. For critically ill children, a clinical officer or a medical doctor was often consulted. Mubende Hospital also had a consultant paediatrician who provided care in the paediatric ward.

Jinja Hospital staffing capacity was different in comparison to the other five hospitals. In addition to clinical officers, intern doctors were responsible for making admission decisions and reviewing admitted children on a daily basis. A team of three consultant paediatricians supervised the interns, reviewed children in the unit, and provided daily support including weekends. In all hospitals, nurses made the admission decision if a clinician was not available.

Most children admitted at all six hospitals were severely ill. Occasionally children with uncomplicated disease were admitted. Possible reasons for admitting children with uncomplicated disease states include, existence of co morbid states warranting admission, self-request for hospitalization, and lack of oral medicines required to treat uncomplicated illness warranting admission for treatment with intravenous medications.

### Quality assurance

Following the initiation of surveillance at the six public hospitals, supportive supervision visits were conducted by the UMSP surveillance team consisting of a physician, laboratory supervisor, and data supervisor. The visits were initially conducted every 2–3 months and were later spaced out to every 4–6 months by 2013. During each visit data quality was assessed and feedback provided. Additional data dissemination workshops were held biannually at each site, and included presentations on data quality, key malaria indicators, morbidity and mortality trends, and treatment practices. Administrative issues, potentially affecting performance at each site, were also discussed at the workshops. Continued support to laboratories, including external quality control, was provided by UMSP and the district health office to ensure adequate staffing, supply of reagents and slides, and adherence to high standards of malaria microscopy. The quality control system used expert microscopists to re-read 50 randomly selected slides and any hospital microscopist with less than 90 % concordance was re-trained. Quality control was carried out monthly at the beginning of the project, but changed to every 6 months after the UMSP team felt that quality was being maintained.

### Data management and statistical analyses

MRFs of discharged patients were collected on a daily basis and entered into an Access database (Microsoft Corporation, Redmond, WA, USA) and transmitted to a central database located in Kampala, Uganda on a monthly basis. Prior to being uploaded to a secure server, data were checked for errors. Stata version 12 was used for data analysis (Stata Corp., College Station, TX, USA).

All children (age <14 years) admitted to the six hospitals from July 2011 through December 2013 were included in the analysis. The following malaria case management indicators were examined: (1) proportion of admitted children with any malaria test result performed; positive or negative, (2) proportion of children prescribed anti-malarial drugs, stratified by positive or negative test result, and (3) type of anti-malarial drug prescribed, stratified by positive or negative result. A patient was reported to have a positive malaria test if any test result [presence of asexual parasitaemia by light microscopy or positive rapid diagnostic test (RDT)] was positive during hospitalization. A patient was reported to have only negative malaria test results if all test results were negative during hospitalization. A patient was defined as having complicated malaria, if had a positive malaria test result and presented with symptoms or signs consistent with WHO definition of severe malaria or had danger signs warranting intravenous treatment [3]. If a patient received more than one anti-malarial drug while in the hospital they were placed in a single category based on the following hierarchy (informed by current national treatment guidelines): (1) intravenous artesunate, (2) intravenous quinine, (3) intramuscular artemether, (4) rectal artesunate, (5) oral artemether–lumefantrine, (6) oral quinine, (7) oral artesunate, (8) oral sulfadoxine–pyrimethamine, and (9) oral chloroquine. A regression model using the Generalized Estimating Equations (GEE) approach was used to estimate associations between covariates of interest and the odds of being prescribed an anti-malarial among children with negative malaria test results, while accounting for repeated measures on the date of admission. Covariates considered included the hospital, age, presence of fever (either reported or objectively measured as ≥38.0 °C), and signs or symptoms of complicated malaria (history of or witnessed convulsions, altered consciousness, tea coloured urine, jaundice, unconsciousness, lethargy, inability to sit, or severe pallor).

## Results

### Overview of malaria diagnostic testing

**Fig. 2:**
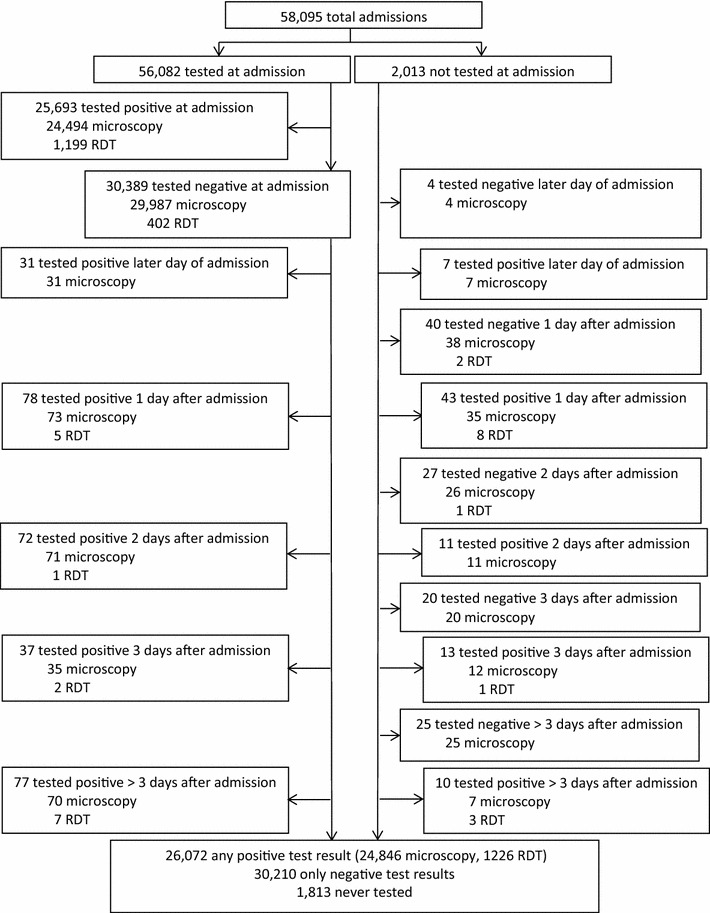
Timing and method of malaria diagnostic testing during hospitalization. Only the first positive and negative test results presented

A total of 58,095 children were admitted to the six hospitals over the 30-month period. Total number of hospital admissions during this period was highest in Jinja (total 20,426; monthly mean 680) and lowest in Kambuga (total 3294; monthly mean 110). Fever was reported or documented in 91.6 % of patients and 49.0 % of patients had signs or symptoms suggestive of complicated malaria. The proportion of children tested for malaria was greater than 95 % at all hospitals and testing rates were consistently high throughout the study period. Among 56,282 patients tested for malaria, 26,072 (46.3 %) tested positive, ranging from 5.9 % in Kabale to 57.3 % in Apac (Table 1). Among those tested for malaria, final test results were based on a RDT for only 1601 patients (2.8 %), with the remainder based on microscopy. Among patients with final test results based on a RDT, 88.8 % were from a single hospital (Tororo). Among patients who tested positive for malaria, only 84 (0.3 %) were first tested after admission (50 within 1 day of admission). Among 30,389 patients who tested negative at admission, 295 (1.1 %) tested positive after an initial negative test (109 within 1 day of admission) (Fig. 2).

**Table 1:** Malaria testing and treatment practices among children admitted to six hospitals: July 2011 to December 2013

Hospital	Total admissions	Tested for malaria (% total)	Test positivity rate (%)	Proportion prescribed an anti-malarial drug (%)	
				Positive test result	Negative test result
Tororo	13,538	12,962 (95.7)	57.3	7291/7421 (98.2)	1154/5541 (20.8)
Jinja	20,426	19,800 (96.9)	46.6	9085/9229 (98.4)	5661/10,571 (53.6)
Kambuga	3294	3248 (98.6)	32.4	998/1052 (94.9)	877/2196 (39.9)
Mubende	10,030	9751 (97.2)	43.4	4077/4234 (96.3)	2958/5517 (53.6)
Apac	6946	6755 (97.3)	57.9	3832/3914 (97.9)	814/2841 (28.7)
Kabale	3861	3766 (97.5)	5.9	200/222 (90.1)	513/3544 (14.5)

### Anti-malarial prescription practices among children with positive test results

Greater than 94 % of children with any positive malaria test result were prescribed an anti-malarial in all hospitals, with the exception of Kabale hospital (90.1 %), the site with the lowest malaria test positivity rate. In Kabale, the practice of prescribing anti-malarials to patients with any positive malaria test decreased significantly in 2013 compared to the previous 18 months (82.6 vs 94.8 %; p = 0.003). In all 22 cases where no anti-malarial was prescribed despite a positive test in Kabale, the positive test was performed at the time of admission.

**Fig. 3:**
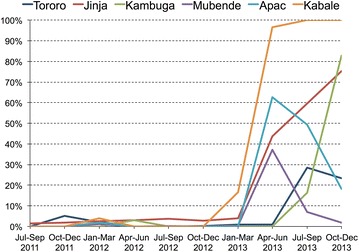
Temporal trends in the proportion of children prescribed intravenous artesunate among those with any positive malaria test result who were prescribed any anti-malarial stratified by study site

Among children with a positive malaria test result who were prescribed an anti-malarial, intravenous quinine was prescribed most frequently (81.9 %), ranging from 52.5 % in Kabale to 87.8 % in Tororo (Table 2). The next most common anti-malarials prescribed were intravenous artesunate (13.2 %), intramuscular artemether (3.0 %), oral artemether-lumefantrine (1.4 %), and oral quinine (0.4 %). Intravenous artesunate was prescribed to fewer than 5 % of children who were prescribed an anti-malarial after a positive malaria test result until April 2013, when the proportion began increasing (Fig. 3). The increase in IV artesunate was not sustained at Apac, Tororo, and Mubende where a decline was observed during the final months of 2013. Overall, in the latter half of 2013, 35.2 % of children with a positive malaria test receiving an anti-malarial were prescribed intravenous artesunate. During this same 6-month period, the proportion of these children who were prescribed intravenous artesunate varied widely by health facility with Kabale (100 %) having the highest proportion, followed by Jinja (67.3 %), Kambuga (43.2 %), Apac (32.6 %), Tororo (26.2 %), and Mubende (4.2 %).

**Table 2:** Anti-malarial drugs prescribed to hospitalized children with positive malaria test results

Anti-malarial type	Hospital					
	Tororo (n = 7291) (%)	Jinja (n = 9085) (%)	Kambuga (n = 998) (%)	Mubende (n = 4077) (%)	Apac (n = 3832) (%)	Kabale (N = 200) (%)
IV quinine	6404 (87.8)	7162 (78.8)	842 (84.4)	3513 (86.2)	2842 (74.2)	105 (52.5)
IV artesunate	457 (6.3)	1578 (17.4)	112 (11.2)	239 (5.9)	915 (23.9)	66 (33.3)
IM artemether	336 (4.6)	262 (2.9)	10 (1.0)	141 (3.5)	5 (0.1)	7 (3.5)
Oral artemether–lumefantrine	87 (1.2)	59 (0.6)	12 (1.2)	125 (3.1)	55 (1.4)	17 (8.5)
Oral quinine	3 (0.04)	16 (0.2)	18 (1.8)	54 (1.3)	8 (0.2)	3 (1.5)
Rectal artesunate	2 (0.03)	1 (0.01)	4 (0.4)	2 (0.04)	7 (0.2)	1 (0.5)
Oral sulfadoxine–pyrimethamine	1 (0.01)	4 (0.04)	0	2 (0.04)	0	1 (0.5)
Oral artesunate	1 (0.01)	3 (0.03)	0	0	0	0
Oral chloroquine	0	0	0	1 (0.02)	0	0

### Anti-malarial prescription practices among children with negative test results

Among the 30,210 children with only negative malaria test results, 11,977 (39.6 %) were prescribed any anti-malarial drugs, ranging from 14.5 % in Kabale to 53.6 % in Jinja and Mubende (Table 1). Anti-malarial treatment practices differed based on malaria test results. Compared to children with any positive test result, children with only negative test results were less likely to be prescribed intravenous quinine (81.9 vs 74.7 %, p < 0.001) or intravenous artesunate (13.2 vs 10.1 %; p < 0.001); and more likely to be prescribed intramuscular artemether (2.9 vs 5.4 %; p < 0.001) and oral artemether–lumefantrine (1.3 vs 7.5 %; p < 0.001).

**Fig. 4:**
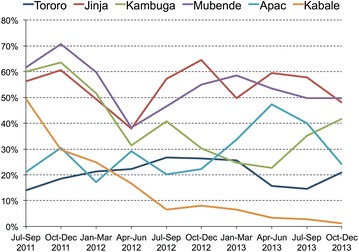
Temporal trends in the proportion of children prescribed anti-malarial drugs among those with only negative malaria test results stratified by study site

The proportion of children with only negative malaria test results who were prescribed any anti-malarial drug during the first 3 months of the study period varied from 13.9 % in Tororo to 61.8 % in Mubende. During the course of the study, trends in prescription practices among children with only negative malaria test results showed a steady decline in Kabale, where 165 of 334 (49.4 %) children were prescribed any anti-malarial drug during the first 3 months of the study, decreasing to only 4 of 337 (1.2 %) children during the last 3 months of the study. However, the other five hospitals showed greater fluctuation and no significant trend in the practice of prescribing any anti-malarial drug to children with only negative malaria test results (Fig. 4).

### Predictors of anti-malarial prescription to children with only negative results

Associations between covariates of interest including the hospital, age, the presence of fever, and clinical findings suggestive of complicated malaria with the odds prescribing any anti-malarial drugs to children with only negative malaria test results are presented in Table 3. The hospital was strongly associated with the odds of anti-malarial prescription among children with only negative malaria test results. Compared to the hospital (Kabale) with the lowest frequency of prescribing anti-malarial drugs (14.5 %), the adjusted odds ratio (aOR) of prescribing an anti-malarial using multivariate analysis ranged from 1.77 (p < 0.001) in Tororo to 7.63 (p < 0.001) in Jinja. Age was also strongly associated with the odds of anti-malarial prescription to children with only negative malaria test results, as children 6–12 months (aOR 3.78, p < 0.001) and greater than 12 months (aOR4.89, p < 0.001) were more likely than children under 6 months of age to be prescribed an anti-malarial. Selected symptoms and physical examination findings were also associated with the odds of being prescribed an anti-malarial drug among children with only negative malaria test results. Children who had fever (as reported by the parent or measured at admission) had a higher odds of being prescribed an anti-malarial compared to those with no fever (43.8 vs 12.2 %, aOR 5.43, p < 0.001). Children with symptoms and physical exam findings suggestive of complicated malaria had a higher odds of being prescribed for an anti-malarial (49.7 vs 29.2 %, aOR 1.98, p < 0.001).

**Table 3:** Covariates associated with prescription of an anti-malarial drug among children with only negative malaria tests results

Covariates	Proportion prescribed an antimalarial (%)	Univariate analysis			Multivariate analysis		
		OR	95 % CI	p value	aOR	95 % CI	p value
Hospital							
Kabale	513/3544 (14.5)		Reference			Reference	
Tororo	1154/5541 (20.8)	1.55	1.39-1.74	<0.001	1.72	1.44-2.04	<0.001
Apac	814/2841 (28.7)	2.37	2.10-2.69	<0.001	2.69	2.23-3.24	<0.001
Kambuga	877/2196 (39.9)	3.93	3.46-4.46	<0.001	3.39	2.88-4.00	<0.001
Jinja	5661/10,571 (53.6)	6.81	6.16-7.54	<0.001	7.45	6.37-8.71	<0.001
Mubende	2958/5517 (53.6)	6.83	6.13-7.60	<0.001	7.19	6.17-8.39	<0.001
Age							
<6 months	692/4062 (17.0)		Reference			Reference	
6–12 months	2440/6272 (38.9)	3.10	2.82–3.41	<0.001	3.82	3.43–4.26	<0.001
≥12 months	8840/19,866 (44.5)	3.90	3.58–4.26	<0.001	4.89	4.45–5.41	<0.001
Fever^a^							
Absent	487/3982 (12.2)		Reference			Reference	
Present	11,490/26,216 (43.8)	5.60	5.08–6.18	<0.001	5.41	4.86–6.02	<0.001
Clinical findings suggestive of complicated malaria^b^							
Absent	4329/14,819 (29.2)		Reference			Reference	
Present	7648/15,391 (49.7)	2.39	2.28–2.51	<0.001	1.94	1.83–2.06	<0.001

Clustering on the day of admission adjusted using GEE

^**a**^History of fever or elevated temperature

^b^History of or witnessed convulsions, altered consciousness, tea coloured urine, jaundice, unconsciousness, lethargy, inability to sit, or severe pallor

## Discussion

Demonstration of the presence of malaria parasites prior to treatment with appropriate anti-malarial medicine has recently been adopted as a global standard of care for malaria case management [15]. Proper diagnosis and treatment of malaria is especially crucial in Ugandan hospitals as a majority of admitted children have fever as well as other symptoms and signs suggestive of complicated malaria. Presented is data on malaria prescription practices from a sentinel site programme, where malaria diagnostic capacity is supported, clinicians receive malaria case management training, and the testing of each admitted child for malaria is encouraged and subject to external quality assurance. Presented data are notable for high rates of malaria diagnostic testing, a recent shift from the prescription of intravenous quinine to intravenous artesunate for malaria, and frequent prescription of anti-malarials to children with a negative malaria test result. The analysis also reveals factors associated with the prescription of anti-malarials to children with only negative malaria test results, findings which will be critical to designing programmes to improve provider compliance with malaria case management guidelines.

Through sustained supervision, adopting a goal of universal testing, and buffering of the supply chain for essential materials required for malaria diagnostic testing, testing rates over 95 % among children admitted to the six public hospitals were achieved. Among children with positive tests results, intravenous quinine was the most commonly prescribed anti-malarial. At the start of the programme, intravenous artesunate, recommended by WHO and the Ministry of Health as first line treatment for complicated malaria in children, was rarely prescribed. However, in the latter half of 2013, a dramatic increase in prescription rates of intravenous artesunate was noted across all sites, though this was not sustained at some sites in subsequent months. The rise in prescription rates of intravenous artesunate corresponded to the actual implementation of the policy change adopted 2 years earlier in Uganda. The rapid decline in prescription rates observed at two sites following the initial surge was attributed to failed reacquisition of intravenous artesunate. These difficulties highlight the challenges of implementing and sustaining new policies in resource-limited settlings like Uganda.

Among children with only negative malaria test results, data showed high rates of prescription of anti-malarials, results comparable to those reported in a similar setting in Tanzania prior to the global recommendation for universal diagnostic testing for malaria [12]. Despite training efforts, only one hospital, located in the lowest transmission setting, demonstrated a decline in this practice. A case for the presumptive treatment in endemic areas in the absence of laboratory confirmation has been argued (especially in the setting of unreliable laboratory diagnosis) [16–20], however this argument becomes less acceptable in low transmission settings where the positive predictive value of clinical diagnosis is low [8]. Furthermore, the practice of withholding anti-malarials from children after a negative malaria test has been shown to be safe and effective in a variety of transmission settings, including hospitalized children [20–23]. Indeed, in our study over 96 % of patients were tested at admission and only 1 % of those who tested negative at admission went on to have a positive test. Additionally, prescription of anti-malarials to hospitalized children with negative test results has been associated with increased risk of mortality, a fact attributed to mismanagement of the true underlying (non-malarial) cause of illness [24–26]. Contrary to expectations, these results show that prescription rates to children with negative test results at the six hospitals did not correlate with reported endemicity levels at respective sites, implying that other factors, such as qualifications, beliefs, and attitudes of health workers were likely in play [18]. Regardless of variation in practice, and for as long as laboratory test results are credible, as was the case in the settings where this study was conducted, prescribing anti-malarials to children with negative test results is wasteful, puts patients at risk of harmful effects of medicines, and may promote drug resistance [12, 16, 27, 28]. Furthermore, amidst procurement and supply challenges, sustaining supply of intravenous artesunate cannot be guaranteed amidst the indiscriminate prescription of anti-malarials [24, 25, 29, 30]. Finally, diagnosing and treating children with negative malaria test results can erode health worker confidence in otherwise effective medicines, miss other serious causes of febrile illness, and contribute to poor outcomes, including a possible increase in mortality [12, 19, 26, 31]. It should be noted that while the rate of anti-malarial prescription to children with a negative malaria test was high at the hospitals involved in this study, it does compare favorably to rates published at other Ugandan hospitals [32, 33], likely reflecting the influence of UMSP training efforts.

Key to lowering the rate of prescription of anti-malarials to children with negative test results will be a better understanding of factors associated with this case management decision, and the results suggest possible reasons for this practice. With limited clinical skills and diagnostic capacity to evaluate patients, clinicians are unable to confirm alternate diagnoses and often disregard negative test results. Presented data support this hypothesis—children presenting with signs and symptoms similar to those of complicated malaria, such as pallor, and histories of fever, convulsions, and passing tea coloured urine were more likely to be prescribed anti-malarials after a negative test than children who did not have these findings. Additional evidence in support of this hypothesis is that the magnitude of association between study sites and anti-malarial prescription among children with negative test results doubled upon regression analysis limited to children with clinical findings suggestive of complicated malaria, as compared to analysis limited to those without clinical findings suggestive of complicated malaria. In addition, qualitative research work has shown that health worker beliefs towards perceived benefits of anti-malarial treatment are often overrated superseding rational clinical judgment. This behaviour is likely influenced by promotion of malaria as an important disease [34], peer pressure and patient demand for anti-malarial drugs which are often available at most health facilities [31, 35, 36], encouraging clinicians to treat malaria even in situations where malaria is unlikely [37]. In as much as prescription of anti-malarial to children with negative results is irrational, circumstances may exist when the practice is justified. For example, it may be reasonable to prescribe anti-malarials to someone who tested negative but was admitted after therapy had already been initiated at another health facility. Additionally, long turnaround times for test results warrant starting therapy before the test results are available. Importantly, the findings demonstrate the importance of repeating a negative test result when in doubt, as even in the study setting a small proportion of children who were initially negative became positive upon repeat testing.

This study had some limitations. First, it is possible that some treatments documented on the MRF were never administered and others were administered without documentation. Additionally, study data came from a malaria sentinel surveillance site programme and may not reflect what is happening at other public hospitals in Uganda. Furthermore, classification of those with malaria was limited to diagnostic testing results and it was not possible to ascertain where patients fell on the spectrum of disease from asymptomatic parasitaemia to complicated malaria. Lastly, data on treatment prior to hospitalization was not captured, which may have influenced some clinicians to prescribe anti-malarials even when test results were negative.

## Conclusions

Demonstrated is that the practice of irrational prescription of anti-malarials remained high at six hospitals serving as malaria sentinel sites. These findings are of concern considering that Uganda changed policy to laboratory based confirmation, and that testing rates were high in the hospitals where the study was done, implying that the situation could be more serious in government hospitals where opportunities for testing, training and supervision are not comparable. Given this, it will continue to be extremely challenging to implement the WHO test and treat policy in Uganda and similar settings [37]. Sustained interventions targeted towards strengthening diagnostic and treatment capacity for malaria and non-malaria febrile disease, and changing health worker beliefs and attitudes towards malaria diagnosis are required. Behaviour change can be achieved through implementation of standard training programmes integrated into vigorous evaluation, supervision, and mentorship systems.
